# Assessment of risk based on variant pathways and establishment of an artificial neural network model of thyroid cancer

**DOI:** 10.1186/s12881-019-0829-4

**Published:** 2019-05-28

**Authors:** Yinlong Zhao, Lingzhi Zhao, Tiezhu Mao, Lili Zhong

**Affiliations:** 1 Department of Nuclear Medicine, The Second Hospital of Jilin University, Changchun, Jilin 130041 People’s Republic of China; 2 Purchasing Center, The Second Hospital of Jilin University, Changchun, Jilin 130041 People’s Republic of China; 3 Department of radiotherapy, The Second Hospital of Jilin University, Changchun, Jilin 130041 People’s Republic of China; 4 Jilin Provincial Key Laboratory on Molecular and Chemical Genetic, The Second Hospital of Jilin University, Changchun, Jilin 130041 People’s Republic of China

**Keywords:** Thyroid cancer, Risk assessment, Variant pathway, Artificial neural network

## Abstract

**Background:**

This study aimed to establish an artificial neural network (ANN) model based on variant pathways to predict the risk of thyroid cancer.

**Methods:**

The RNASeq data of 482 thyroid cancer samples were downloaded from the TCGA database. The samples were divided into low-risk and high-risk groups, followed by identification of differentially expressed genes (DEGs). Co-expression analysis and pathway enrichment analysis were then performed. The variant pathways were screened according to the functional deviation score of each pathway, and an ANN model was established. Finally, the efficiency of the ANN model for risk assessment was validated by survival analysis and analysis of an independent microarray dataset (GSE34289) for thyroid cancer.

**Results:**

In total, 190 DEGs (85 up-regulated and 105 down-regulated) were identified between the low-risk and high-risk groups. Ten risk-related variant pathways were identified between the low-risk and high-risk groups, which were related to inflammatory and immune responses. Based on these variant pathways, an ANN model was built, consisting of an input layer, two hidden layers, and an output layer, corresponding to 15, 8, 5, and 1 neuron, respectively. Survival analysis showed that this model could effectively distinguish the samples with different risks. Analysis of microarray dataset GSE34289 showed that the accuracy of this model for predicating low-risk and high-risk samples was 77.5 and 86.0%, respectively.

**Conclusions:**

This study suggests that the ANN model based on variant pathways can be used for effectively evaluating the risk of thyroid cancer.

**Electronic supplementary material:**

The online version of this article (10.1186/s12881-019-0829-4) contains supplementary material, which is available to authorized users.

## Background

Thyroid cancer is the most prevalent endocrine malignant cancer, with a steadily increasing incidence rate over the past several years [1]. Differentiated thyroid cancer comprises the majority (> 90%) of all thyroid cancers, including papillary and follicular cancer [2, 3]. The main treatments for thyroid cancer are surgery, TSH suppressive treatment, and radioactive iodine (RAI) ablation therapy, and the average overall 5-year survival rate is up to 97.7% [4]. However, approximately 10–20% patients lose their life due to the recurrence or progression of thyroid cancer [5]. Therefore, exploring effective approaches is of great importance for the diagnosis of the risk of thyroid cancer.

During the past several decades, multiple computer-aided diagnostic models have been used for predicting the risk of a variety of cancers, such as logistic regression, Cox proportional hazard model, and decision trees [6–8]. Artificial neural networks (ANNs) represent a more recent approach for risk assessment of multiple diseases, including Parkinson’s disease [9], cardiovascular autonomic dysfunction [10], metabolic disorders [11], and various cancers [12–14]. Notably, ANN-based exploration of gene-nutrient interactions in folate and xenobiotic metabolic pathways can be used for investigating how micronutrients regulate susceptibility to breast cancer [15]. In thyroid cancer, Notch signaling is found to regulate tumor growth [16]. PI3K/Akt signaling pathway is considered a key mechanism to regulate the tumor-suppressive effects of metallothionein 1G in thyroid cancer [17]. The sonic hedgehog signaling pathway can induce snail expression and thereby control the self-renewal of stem cells in anaplastic thyroid cancer [18]. Although many signaling pathways have been found to be involved in thyroid cancer, studies on the use of ANN-based pathways for predicting thyroid cancer risk are rare.

In this study, we downloaded the RNASeq data for thyroid cancer samples with different cancer risks from The Cancer Genome Atlas (TCGA) database. Differentially expressed genes (DEGs) were identified between low-risk and high-risk groups, followed by co-expression analysis and pathway enrichment analysis. The variant pathways between the low-risk and high-risk groups were screened according to functional deviation score of each enriched pathway and an ANN model was established. Moreover, combined with the survival data for these samples, the efficiency of ANN model for risk assessment was validated by survival analysis and an independent microarray dataset of thyroid cancer, GSE34289. Microarray dataset GSE34289 has been utilized to build a gene-expression classifier for improving preoperative risk assessment [19]. Our study results should provide new insights for predicting the risk of thyroid cancer.

## Methods

### Data source

The RNASeq data of thyroid cancer, including 482 thyroid cancer samples, were downloaded from TCGA (https://portal.gdc.cancer.gov/) in April 2017, based on the thca_tcga_pub_rna_seq_v2_mrna platform. The clinical information of thyroid cancer samples downloaded from the TCGA database was shown in Additional file 1: Table S1. The downloaded data had been preprocessed.

### Data reconstruction and grouping

Based on the clinical data and information, the thyroid cancer samples downloaded from TCGA database were divided into low-risk (including T0, T1, N0, and M0) and high-risk (including T2, N1, M1, and above stages) groups according to the International Union Against Cancer (UICC) tumor–node–metastasis (TNM) classification. Finally, 114 high-risk samples and 368 low-risk samples were distinguished.

### Data normalization and identification of DEGs

To eliminate the inherent expression differences between genes, the low-risk group was used as control to normalize the expression value of all samples using Z-score transformation [20]. The expression values with more than 2.5-fold change in terms of the standard deviation were defined as abnormal and subjected to correction. The DEGs between high-risk samples and low-risk samples were then identified using the *limma* package (version 3.10.3, http://www.bioconductor.org/packages/2.9/bioc/html/limma.html) [21]. *P* < 0.05 and coefficient of variance (CV) > 33.8 or < − 35.1 were used as the cut-off values to identify the DEGs.

### Coexpression network analysis

The coexpression analysis of different genes between low-risk and high-risk samples was evaluated using Pearson correlation coefficient. R > 0.5 was identified as positive correlation, whereas R < − 0.5 indicated negative correlation. In both low-risk and high-risk samples, the coexpressed genes were defined as stable pairs, whereas the coexpressed genes found only in one of the groups were considered specific pairs. The stable pairs were recognized as the key genes with important functions, whose coexpression was not markedly affected by environment or disease stimulation. Nevertheless, the coexpression of specific pairs would change during tumor initiation and progression, which could be used for evaluating the cancer risk. After identifying the stable and specific pairs, unsupervised hierarchical clustering analysis [22] for the genes in these pairs was performed using the *heatmap2* package in R [23], for distinguishing the samples with different cancer risks. In addition, based on the coexpression relationships, the respective gene coexpression networks under the low-risk and high-risk status were constructed using Cytoscape 3.4.0 [24]. Two topological properties, including average shortest path (ASLP) and degree distribution, were then analyzed to measure the connectivity of the network.

### Pathway enrichment analysis and pathway deviation score

To further analyze the biological functions of the genes in stable pairs and specific pairs, KEGG pathway enrichment analysis was carried out using Fisher’s exact test in the Database for annotation, visualization, and integrated discovery (DAVID) online tool [13]. A value of *P* < 0.05 was used as the cut-off value. As these DEGs were associated with the risk of cancer, these significantly enriched pathways might be used for the evaluation of gene functions related to cancer risk. Based on the expression of DEGs in each sample, the functional deviation score for significantly enriched pathways was calculated using the Euclidean distance algorithm according to the following formula:

$$ \mathrm{score}\left(\mathrm{P}\right)=\frac{\sqrt{\sum_{\mathrm{i}=1}^{\mathrm{n}}{\left(\mathrm{Gi}-\mathrm{mean}\right)}^2}}{\mathrm{n}} $$

In the formula, n represents the number of enriched genes, Gi represents the expression of a single gene, and mean is the mean value of Gi in the low-risk group. High scores indicate a marked pathway deviation from normal levels of the low-risk group, whereas low scores indicate that the pathway expression levels were close to the normal levels of the low-risk group. Using this method, the differing pathways between the low-risk and high-risk groups were screened out using Student’s t-test. *P* < 0.01 was used as the cut-off value for significance.

### Establishment of ANN model based on the variant pathways

Based on the DEGs and functional pathway analysis, an ANN model was established using the supervised classification method, with multilayer perceptron (MLP) algorithm [25]. Meanwhile, the ROC curve was drawn with five-time cross validation to evaluate the classification efficiency of ANN model. Meanwhile, logistic regression as a reference was also analyzed to evaluate the efficiency of ANN model, based on variant pathways for risk assessment of thyroid cancer.

### Validation with survival analysis

Using the above ANN model, 482 thyroid cancer samples obtained from the TCGA database was classified into two groups with different cancer risks. Using the survival data for these samples, survival analysis for the low-risk and high-risk groups was conducted using the *survival* package in R, and significant differences in survival times between two groups were determined using log-rank test. To further validate the efficiency of the ANN model for risk assessment, an independent dataset, with accession number GSE34289 deposited by Alexander et al. [19], was downloaded from the Gene Expression Omnibus (GEO, https://www.ncbi.nlm.nih.gov/geo/query/acc.cgi), generated on the GPL14961 platform ([HuEx-1_0-st] Affymetrix Human Exon 1.0 ST Array [transcript (gene) version]). A total of 318 thyroid cancer samples were used in this study, including 40 benign, 233 indeterminate, and 45 malignant samples. The clinical information of thyroid cancer samples downloaded from the GEO database was shown in Additional file 2: Table S2. The downloaded data had been preprocessed. The risk for these 318 thyroid cancer samples was then predicted using this ANN model.

## Results

### Identification of DEGs in thyroid cancer

Based on the criteria of *P* < 0.05 and CV > 33.8 or CV < − 35.1, a total of 190 DEGs were screened out between low-risk and high risk groups, including 85 up- and 105 down-regulated genes.

### Co-expression network analysis

**Fig. 1:**
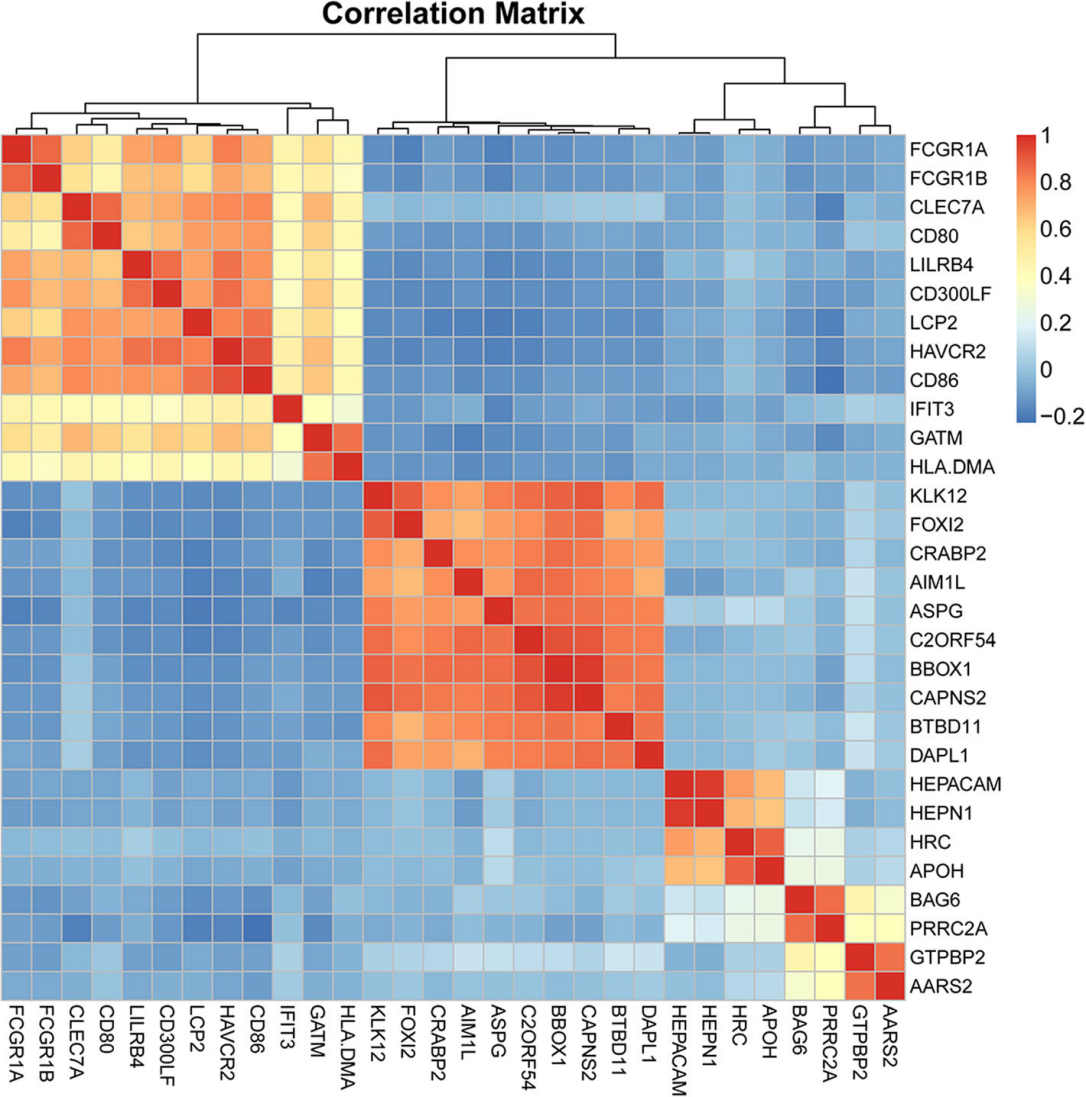
Heat map of top 30 differentially expressed genes (DEGs) with the highest correlation scores. The labels on the abscissa and the longitudinal axis represent the DEGs. Red squares indicate positive correlation, whereas blue squares indicate negative correlation. Deeper colors indicate stronger correlation scores

**Fig. 2:**
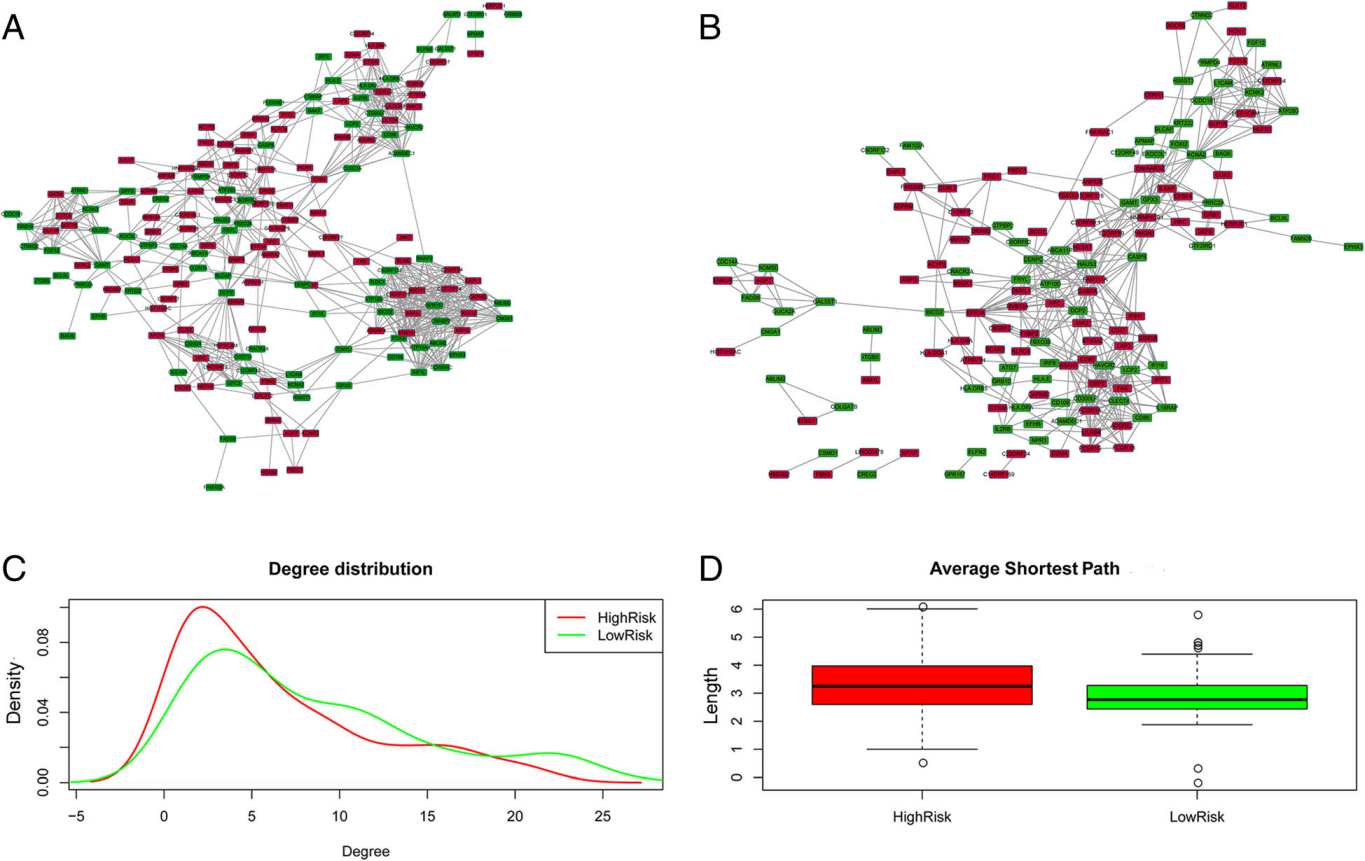
Co-expression networks for low-risk group (**a**) and high-risk group (**b**), and their topological properties, including degree distribution (**c**) and the average shortest path length (**d**). Red nodes indicate up-regulated genes, whereas green nodes indicate down-regulated genes

**Fig. 3:**
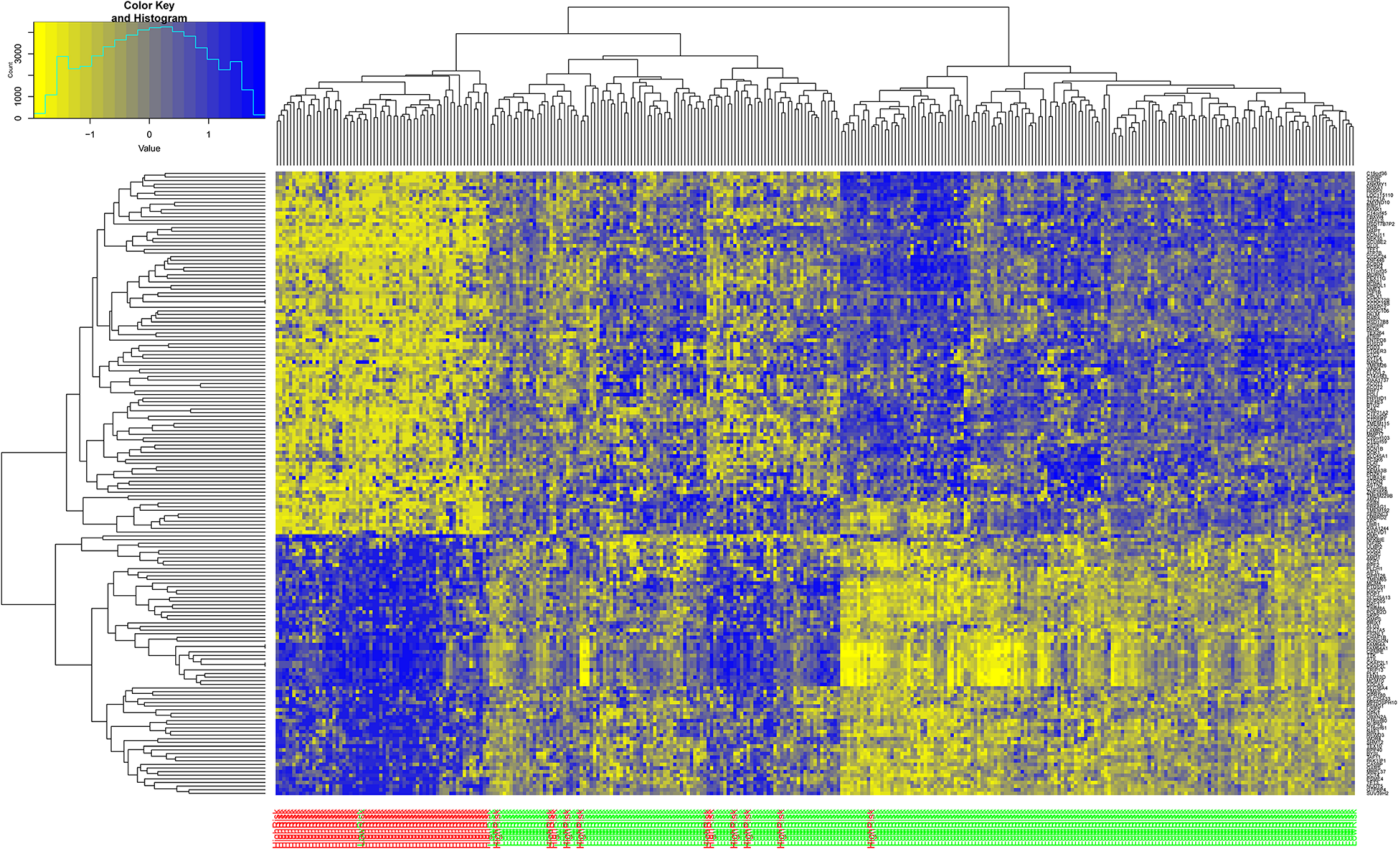
Supervised hierarchical clustering analysis for coexpressed DEGs. The labels on the abscissa below the plot represent samples, and the markings above the plot represent the clustering of samples. The markings on the longitudinal axis represent the clustering of coexpressed DEGs

Pearson correlation coefficient was used to evaluate the correlation between a given pair of DEGs. Figure 1 displays the heat map of the top 30 risk-related DEGs with the highest correlation. According to the coexpression relationships between the gene pairs, the respective gene coexpression networks for the DEGs between low-risk and high-risk groups were constructed (Fig. 2a and b). From the topological perspective of coexpression networks, we found that these risk-related DEGs had a high degree of aggregation and central. Compared with the low-risk group, the high-risk group had more low-degree nodes (Fig. 2c), suggesting that, with increasing risk of thyroid cancer, the network node degree distribution gradually decreased and the association between the genes was gradually lost. Meanwhile, compared with the low-risk group, the average shortest path length in the high-risk group was higher, indicating a decreased capacity of information transfer through the network with increasing risk of thyroid cancer (Fig. 2d). In addition, supervised hierarchical clustering analysis was performed for these coexpressed DEGs, and the results showed that the samples with different cancer risk could be successfully distinguished (Fig. 3).

### Variant pathway analysis and establishment of ANN model for risk assessment

**Fig. 4:**
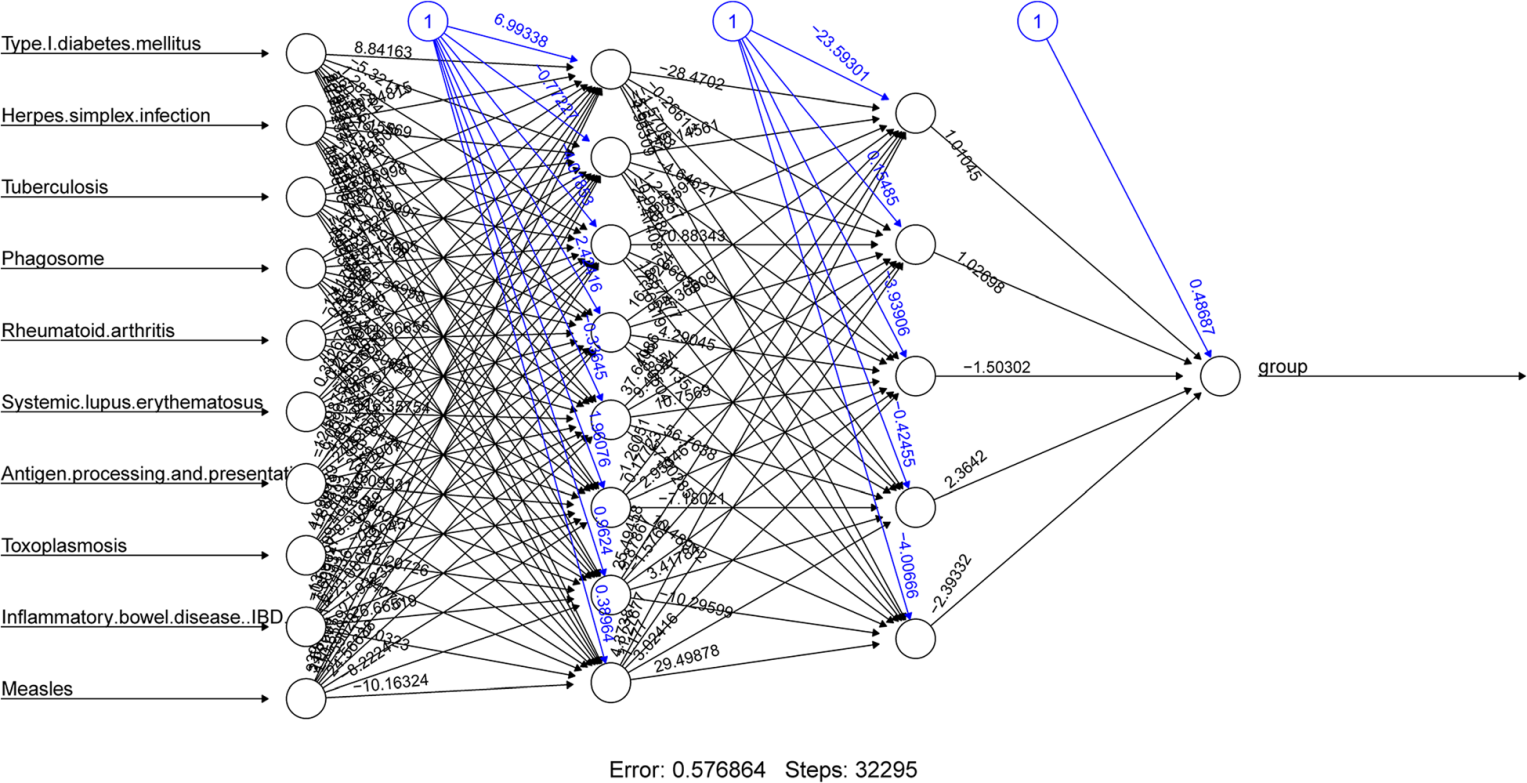
Artificial neural network (ANN) based on 10 significant pathways, consisting of an input layer, two hidden layers, and an output layer, corresponding to 15, 8, 5, and 1 neuron, respectively. The circles represent neurons, while the lines between the circles represent connections. The black lines represent the connections between the neurons, while the blue lines represent the weights

**Fig. 5:**
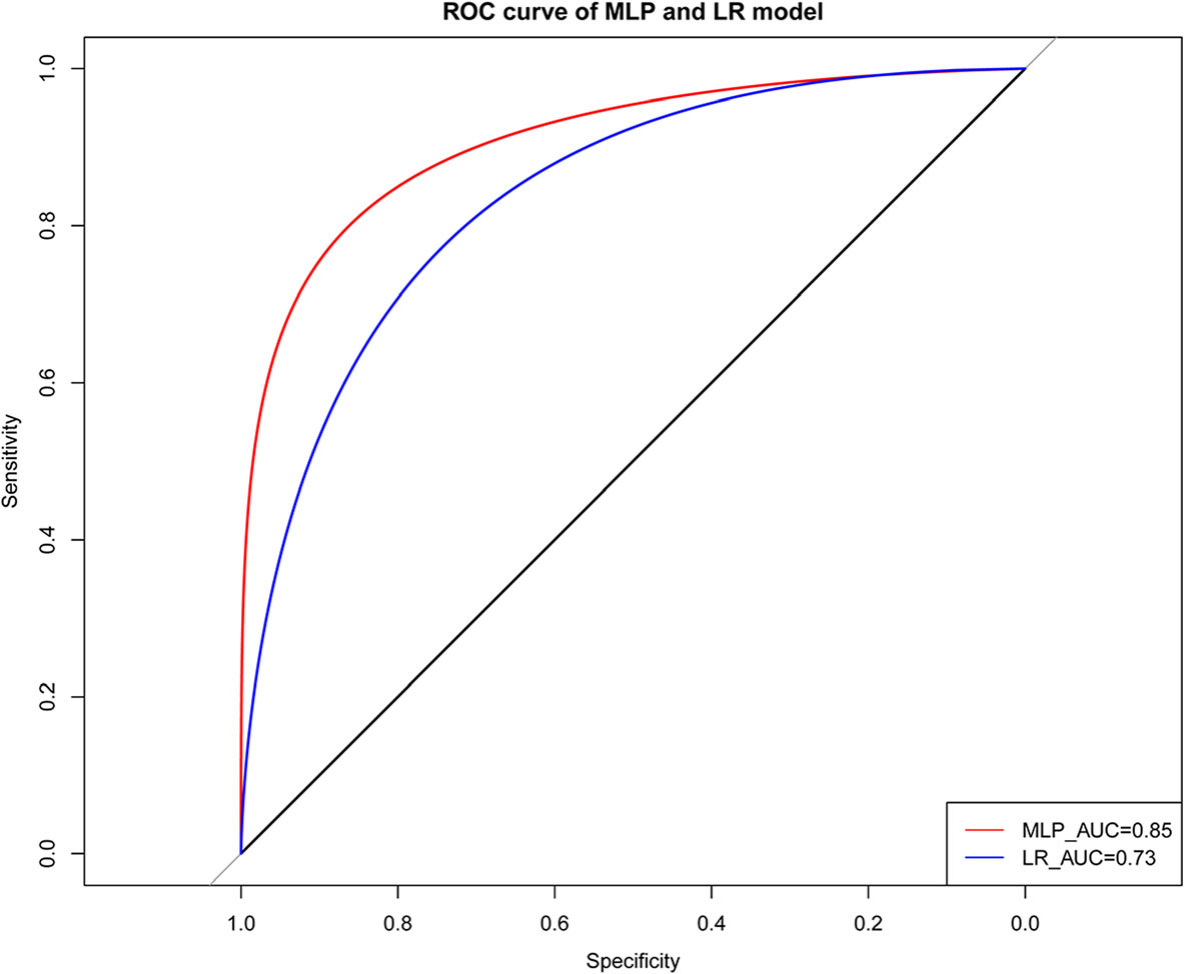
ROC curve of the ANN model (red line) and the logistic regression model (blue line)

KEGG pathway enrichment analysis for risk-related DEGs revealed 21 significantly enriched pathways, including those for Graft-versus-host disease, Allograft rejection, Type I diabetes mellitus, Autoimmune thyroid disease, Viral myocarditis, and Herpes simplex infection (Table 1). In order to quantify these significantly enriched pathways, the functional deviation score for each pathway was calculated for the identification of variant pathways associated with cancer risk. Using Student’s t-test with a significance threshold of *P* < 0.01, 10 risk-related variant pathways with significant differences between low-risk and high-risk groups were identified, including those for Measles, Antigen processing and presentation, Rheumatoid arthritis, Phagosome, Systemic lupus erythematosus, Herpes simplex infection, Inflammatory bowel disease IBD, Tuberculosis, Type I diabetes mellitus, and Toxoplasmosis (Table 2), most of which were related to inflammatory and immune responses. An ANN model for risk assessment was then constructed, for which the above 10 risk-related pathways served as the input variables (Fig. 4). This ANN model consisted of an input layer, two hidden layers, and an output layer, respectively, corresponding to 15, 8, 5, and 1 neuron, respectively. To assess the performance of this ANN model, ROC curves for this model and logistic regression model (control) were prepared (Fig. 5). We found that the area under the receiver operating curve (AUC) for the ANN model and logistic regression model was 0.85 and 0.73, respectively, indicating that the ANN model based on variant pathways had a better prediction accuracy for risk assessment.

**Table 1:** KEGG pathway enrichment analysis for differentially expressed genes

Term	Enrichment score	Count	*P* value	Genes
Graft-versus-host disease	23.93073593	8	2.52E-08	CD86, CD80, HLA-DRB5, FAS, HLA-E, HLA-DMA, HLA-DQA1, HLA-DRA
Allograft rejection	21.34362934	8	5.89E-08	CD86, CD80, HLA-DRB5, FAS, HLA-E, HLA-DMA, HLA-DQA1, HLA-DRA
Type I diabetes mellitus	18.80272109	8	1.48E-07	CD86, CD80, HLA-DRB5, FAS, HLA-E, HLA-DMA, HLA-DQA1, HLA-DRA
Autoimmune thyroid disease	15.18681319	8	6.80E-07	CD86, CD80, HLA-DRB5, FAS, HLA-E, HLA-DMA, HLA-DQA1, HLA-DRA
Viral myocarditis	13.85463659	8	1.29E-06	CD86, CD80, CASP8, HLA-DRB5, HLA-E, HLA-DMA, HLA-DQA1, HLA-DRA
Herpes simplex infection	6.473067916	12	1.59E-06	DDX58, HMGN1, IFIH1, GTF2IRD1, CASP8, HLA-DRB5, JAK2, FAS, HLA-E, HLA-DMA, HLA-DQA1, HLA-DRA
Tuberculosis	5.577078289	10	5.85E-05	FCGR1A, CASP8, HLA-DRB5, FCER1G, ATP6V1H, JAK2, CLEC7A, HLA-DMA, HLA-DQA1, HLA-DRA
Cell adhesion molecules (CAMs)	6.256539235	9	7.58E-05	CLDN16, CD86, CD80, HLA-DRB5, L1CAM, HLA-E, HLA-DMA, HLA-DQA1, HLA-DRA
Intestinal immune network for IgA production	12.60182371	6	9.50E-05	CD86, CD80, HLA-DRB5, HLA-DMA, HLA-DQA1, HLA-DRA
Phagosome	5.806722689	9	1.28E-04	FCGR1A, HLA-DRB5, ITGB5, ATP6V1H, CLEC7A, HLA-E, HLA-DMA, HLA-DQA1, HLA-DRA
Asthma	16.45238095	5	2.05E-04	HLA-DRB5, FCER1G, HLA-DMA, HLA-DQA1, HLA-DRA
Rheumatoid arthritis	7.852272727	7	2.26E-04	CD86, CD80, HLA-DRB5, ATP6V1H, HLA-DMA, HLA-DQA1, HLA-DRA
Influenza A	5.10591133	9	3.11E-04	DDX58, IFIH1, HLA-DRB5, JAK2, CPSF4, FAS, HLA-DMA, HLA-DQA1, HLA-DRA
Systemic lupus erythematosus	5.893390192	8	3.50E-04	HIST1H2AC, CD86, CD80, FCGR1A, HLA-DRB5, HLA-DMA, HLA-DQA1, HLA-DRA
Leishmaniasis	8.342052314	6	6.70E-04	FCGR1A, HLA-DRB5, JAK2, HLA-DMA, HLA-DQA1, HLA-DRA
Antigen processing and presentation	7.793233083	6	9.15E-04	KLRC4, HLA-DRB5, HLA-E, HLA-DMA, HLA-DQA1, HLA-DRA
Toxoplasmosis	5.855932203	7	0.001089	CASP8, HLA-DRB5, JAK2, BIRC3, HLA-DMA, HLA-DQA1, HLA-DRA
*Staphylococcus aureus* infection	9.14021164	5	0.001978	FCGR1A, HLA-DRB5, HLA-DMA, HLA-DQA1, HLA-DRA
Inflammatory bowel disease (IBD)	7.712053571	5	0.003688	IL18RAP, HLA-DRB5, HLA-DMA, HLA-DQA1, HLA-DRA
HTLV-I infection	2.69921875	7	0.04181	IL2RB, HLA-DRB5, HLA-E, HLA-DMA, HLA-DQA1, HLA-DRA, APC
Measles	3.711063373	5	0.043409	DDX58, IL2RB, IFIH1, JAK2, FAS

**Table 2:** Selection of risk-related pathways

Pathway	*P* value_	Genes
Measles	2.03E-29	DDX58, IL2RB, IFIH1, JAK2, FAS
Antigen processing and presentation	7.42E-19	KLRC4, HLA-DRB5, HLA-E, HLA-DMA, HLA-DQA1, HLA-DRA
Rheumatoid arthritis	1.70E-13	CD86, CD80, HLA-DRB5, ATP6V1H, HLA-DMA, HLA-DQA1, HLA-DRA
Phagosome	5.84E-12	FCGR1A, HLA-DRB5, ITGB5, ATP6V1H, CLEC7A, HLA-E, HLA-DMA, HLA-DQA1, HLA-DRA
Systemic lupus erythematosus	1.31E-11	HIST1H2AC, CD86, CD80, FCGR1A, HLA-DRB5, HLA-DMA, HLA-DQA1, HLA-DRA
Herpes simplex infection	1.18E-06	DDX58, HMGN1, IFIH1, GTF2IRD1, CASP8, HLA-DRB5, JAK2, FAS, HLA-E, HLA-DMA, HLA-DQA1, HLA-DRA
Inflammatory bowel disease (IBD)	1.14E-05	IL18RAP, HLA-DRB5, HLA-DMA, HLA-DQA1, HLA-DRA
Tuberculosis	0.000357276	FCGR1A, CASP8, HLA-DRB5, FCER1G, ATP6V1H, JAK2, CLEC7A, HLA-DMA, HLA-DQA1, HLA-DRA
Type I diabetes mellitus	0.001284502	CD86, CD80, HLA-DRB5, FAS, HLA-E, HLA-DMA, HLA-DQA1, HLA-DRA
Toxoplasmosis	0.001643	CASP8, HLA-DRB5, JAK2, BIRC3, HLA-DMA, HLA-DQA1, HLA-DRA

### Survival analysis validation

**Fig. 6:**
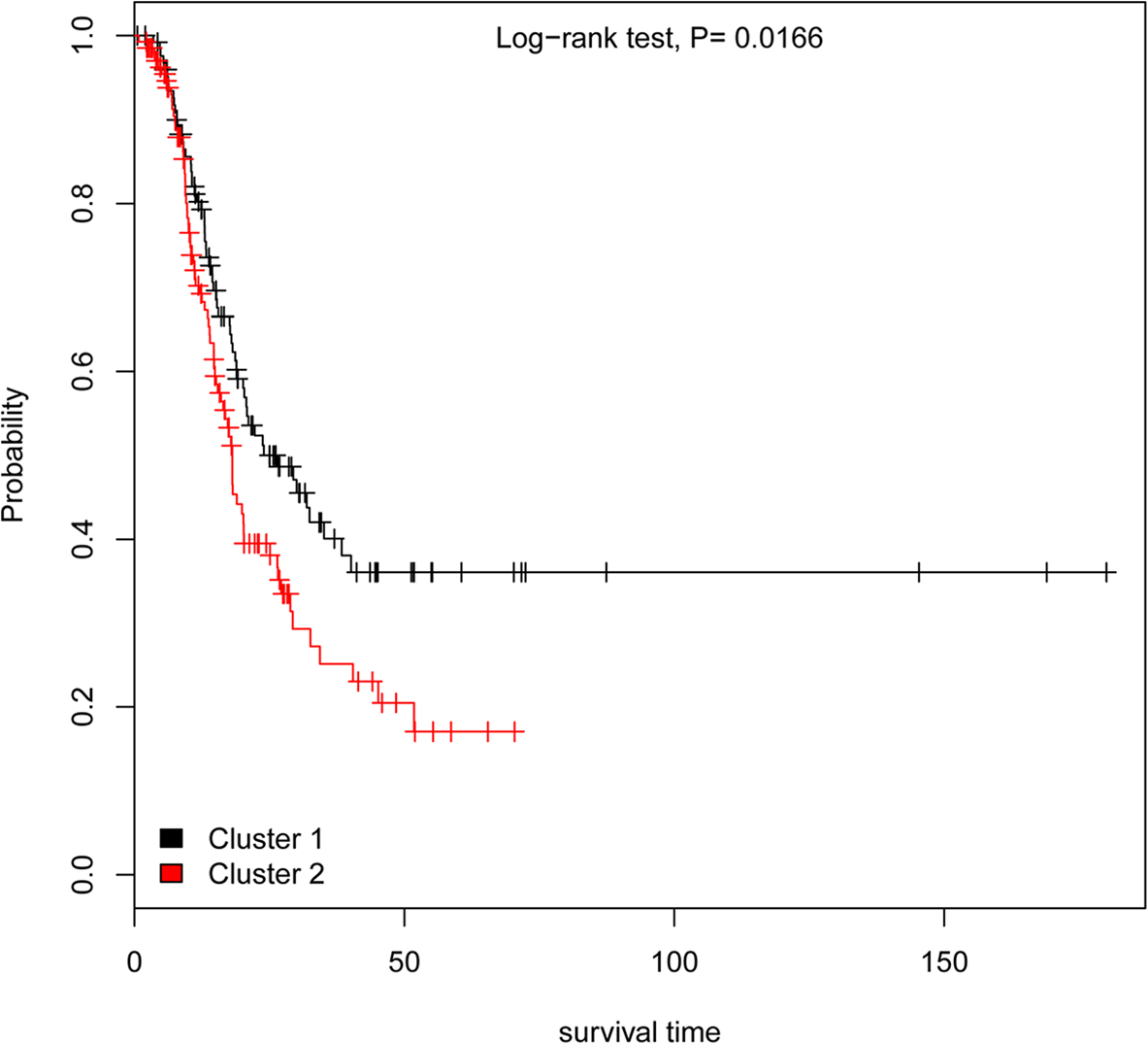
Survival curve of cluster 1 and cluster 2, based on the ANN model. cluster1 represents the low-risk group, whereas cluster 2 represents the high-risk group

The 482 thyroid cancer samples were classified into low-risk and high-risk groups using ANN model. The results of survival analysis showed that the survival time of thyroid cancer samples in the low-risk group was significantly greater than that of the high-risk group samples (*P* = 0.0166, Fig. 6), indicating that this model could effectively distinguish the samples with different risks and yielded accurate predictions for the risk of thyroid cancer. Furthermore, the efficiency of this model was validated using the microarray dataset GSE34289, containing 318 samples representing different stages of thyroid cancer (benign, indeterminate, and malignant). The results showed that the accuracy of this model for low-risk (benign) samples and high-risk (indeterminate and malignant) samples was 77.5 and 86.0%, respectively.

## Discussion

In this study, we developed an ANN model for thyroid cancer, using 10 significantly enriched pathways related to inflammatory and immune responses. Analysis using the survival data of these samples showed that this model could effectively distinguish the samples with different risks. Analysis of microarray dataset GSE34289 showed that the accuracy of this model for predicting low-risk and high-risk samples was 77.5 and 86.0%, respectively. These findings highlight the efficiency of ANN in predicting the risk of thyroid cancer and merit further discussion.

One of the important findings of this study was that 10 variant pathways were identified to establish an ANN model, all of which were related to inflammatory and immune responses. Approximately 15–20% of human tumors are attributed to infection-driven inflammations, and two interrelated pathways have been reported as the link inflammation and cancer [26]. The incidence of thyroid cancer is increased in autoimmune thyroid diseases, and an inflammatory cell infiltrate is often observed, which contributes to the development of thyroid cancer [27]. In addition, the elevated serum concentration of antithyroglobulin antibody and TSH ≥ 1 μIU/ml in patients with Hashimoto’s thyroiditis (a common autoimmune disease) have been confirmed as independent predictors for thyroid cancer [28]. An immunological association has also been reported between Hashimoto’s thyroiditis and thyroid cancer [29], and the pathology of Hashimoto’s thyroiditis can increase the risk of thyroid cancer [30]. The inflammatory microenvironment is shown to play a crucial role in thyroid carcinogenesis [31]. Considering the key roles of inflammatory and immune responses in the development of thyroid cancer, we speculate that these variant pathways may be associated with the risk of thyroid cancer.

Furthermore, ANN and logistic regression model are the most accepted type of models in biomedicine [32–34]. ANN model is considered to be better suited than logistic-regression-based model for predicting outcomes [35]. Consistent with this finding, the ANN model developed here had a higher AUC than the logistic regression model, highlighting the better risk assessment accuracy of the ANN model based on variant pathways. Moreover, the results of survival analysis showed that the survival time of thyroid cancer samples in the low-risk group was greater than that of the high-risk group samples, indicating that this model could effectively distinguish the samples with different risk and was accurate for predicting the risk of thyroid cancer. Furthermore, based on the analysis of microarray dataset GSE34289, the accuracy of this model for low-risk (benign) samples and high-risk (indeterminate and malignant) samples was computed as 77.5 and 86.0%, respectively. These data further confirm the high accuracy of ANN model for the prediction of disease risk, as reported previously [36]. Nevertheless, the performance of the prediction model still needs to be verified through comparison with multiple computer-aided diagnostic models.

## Conclusion

In conclusion, this study suggests that the ANN model based on variant pathways could be used to evaluate the risk of thyroid cancer. With this model, we can identify the patients with a high risk of thyroid cancer, and the model-predicted risk probability would be helpful for clinicians in guiding the management and prevention of cancer high-risk patients.

## Additional files

Additional file 1: **Table S1.** The clinical information of thyroid cancer samples downloaded from the Cancer Genome Atlas (TCGA) database. (XLSX 136 kb)

Additional file 2: **Table S2.** The clinical information of thyroid cancer samples downloaded from the GEO database. (XLSX 25 kb)

## Data Availability

The microarray data GSE34289 was downloaded from the Gene Expression Omnibus (GEO, https://www.ncbi.nlm.nih.gov/geo/query/acc.cgi).

## Abbreviations

ANN: Artificial neural network
ASLP: Average shortest path
CV: Coefficient of variance
DAVID: Database for annotation, visualization, and integrated discovery
DEGs: Differentially expressed genes
MLP: Multilayer perceptron
TCGA: The Cancer Genome Atlas
TNM: Tumor–node–metastasis
UICC: International Union Against Cancer

## References

[1] Davies L, Welch H (2006). Increasing incidence of thyroid cancer in the United States, 1973-2002. JAMA.

[2] Davies L, Welch H (2014). Current thyroid cancer trends in the United States. JAMA Otolaryngol Head Neck Surg.

[3] Lubitz CC, Sosa JA (2016). The changing landscape of papillary thyroid cancer: epidemiology, management, and the implications for patients. Cancer.

[4] Haugen BR, Alexander EK, Bible KC, Doherty GM, Mandel SJ, Nikiforov YE, Pacini F, Randolph GW, Sawka AM, Schlumberger M (2016). 2015 American Thyroid Association management guidelines for adult patients with thyroid nodules and differentiated thyroid Cancer: the American Thyroid Association guidelines task force on thyroid nodules and differentiated thyroid Cancer. Thyroid.

[5] Hollenbeak CS, Boltz MM, Schaefer EW, Saunders BD, Goldenberg D (2013). Recurrence of differentiated thyroid cancer in the elderly. Eur J Endocrinol.

[6] Ayer T, Chhatwal J, Alagoz O, Shavlik J, Kahn CE, Burnside ES: Comparison of Artificial Neural Network and Logistic Regression Model for Breast Cancer Risk Prediction. In: Radiological Society of North America 2008 Scientific Assembly and Meeting. 10.1148/rg.301095057 PMC3709515 19901087

[7] Zhu L, Luo W, Su M, Wei H, Wei J, Zhang X, Zou C: Comparison between artificial neural network and Cox regression model in predicting the survival rate of gastric cancer patients. 2013, 1(5):757–760. 10.3892/br.2013.140 PMC3917700 24649024

[8] Bartfay E, Mackillop WJ, Pater JL (2006). Comparing the predictive value of neural network models to logistic regression models on the risk of death for small-cell lung cancer patients. Eur J Cancer Care (Engl).

[9] Sharma RK, Gupta AK (2015). Voice analysis for Telediagnosis of Parkinson disease using artificial neural networks and support vector machines. Int J Intell Syst Technol Appl.

[10] Tang ZH, Liu J, Zeng F, Li Z, Yu X, Zhou L (2013). Comparison of prediction model for cardiovascular autonomic dysfunction using artificial neural network and logistic regression analysis. PLoS One.

[11] Hirose H, Takayama T, Hozawa S, Hibi T, Saito I (2011). Prediction of metabolic syndrome using artificial neural network system based on clinical data including insulin resistance index and serum adiponectin. Comp Biol Med.

[12] Bertolaccini L, Solli P, Pardolesi A, Pasini A (2017). An overview of the use of artificial neural networks in lung cancer research. J Thorac Dis.

[13] Cancilla JC, Wierzchoś K, Shehadeh N, Haick H, Leja M, Torrecilla JS: Artificial neural networks in the determination of different types of cancer. In: International Symposium on Profiling*:* 2015.

[14] Jerez JM, Franco L, Alba E, Llombartcussac A, Lluch A, Ribelles N, Munárriz B, Martín M (2005). Improvement of breast cancer relapse prediction in high risk intervals using artificial neural networks. Breast Cancer Res Treat.

[15] Naushad SM, Ramaiah MJ, Pavithrakumari M, Jayapriya J, Hussain T, Alrokayan SA, Gottumukkala SR, Digumarti R, Kutala VK (2016). Artificial neural network-based exploration of gene-nutrient interactions in folate and xenobiotic metabolic pathways that modulate susceptibility to breast cancer. Gene.

[16] Yamashita AS, Geraldo MV, Fuziwara CS, Kulcsar MA, Friguglietti CU, Da CR, Baia GS, Kimura ET (2013). Notch pathway is activated by MAPK signaling and influences papillary thyroid cancer proliferation. Transl Oncol.

[17] Fu J, Lv H, Guan H, Ma X, Ji M, He N, Shi B, Hou P (2013). Metallothionein 1G functions as a tumor suppressor in thyroid cancer through modulating the PI3K/Akt signaling pathway. BMC Cancer.

[18] Heiden KB, Williamson AJ, Doscas ME, Ye J, Wang Y, Liu D, Xing M, Prinz RA, Xu X (2014). The sonic hedgehog signaling pathway maintains the cancer stem cell self-renewal of anaplastic thyroid cancer by inducing snail expression. J Clin Endocrinol Metab.

[19] Alexander EK, Kennedy GC, Baloch ZW, Cibas ES, Chudova D, Diggans J, Friedman L, Kloos RT, LiVolsi VA, Mandel SJ (2012). Preoperative diagnosis of benign thyroid nodules with indeterminate cytology. N Engl J Med.

[20] Cheadle C, Vawter MP, Freed WJ, Becker KG (2003). Analysis of microarray data using Z score transformation. J Mol Diagn.

[21] Smyth GK: Limma: linear models for microarray data, in Bioinformatics and computational biology solutions using R and Bioconductor. Springer 2005:397–420.

[22] Sturn A, Quackenbush J, Trajanoski Z (2002). Genesis: cluster analysis of microarray data. Bioinformatics.

[23] Warnes GR, Bolker B, Bonebakker L, Gentleman R, Liaw WHA, Lumley T, Maechler M, Magnusson A, Moeller S, Schwartz M. Gplots: various R programming tools for plotting data. R package version 2.17. 0. Computer software. Available online at: https://cran.r-project.org/web/packages/gplots/index.html.

[24] Shannon P, Markiel A, Ozier O, Baliga NS, Wang JT, Ramage D, Amin N, Schwikowski B, Ideker T (2003). Cytoscape: a software environment for integrated models of biomolecular interaction networks. Genome Res.

[25] Shimray BA, Singh KM, Khelchandra T, Mehta RK: Ranking of sites for installation of hydropower plant using MLP neural network trained with GA: a MADM approach. Computational Intelligence & Neuroscience 2017, 2017. 10.1155/2017/4152140 PMC5346385 28331490

[26] Allavena P, Garlanda C, Borrello MG, Sica A, Mantovani A (2008). Pathways connecting inflammation and cancer. Curr Opin Genet Dev.

[27] Guarino V, Castellone MD, Avilla E, Melillo RM (2010). Thyroid cancer and inflammation. Mol Cell Endocrinol.

[28] Azizi G, Keller J, Lewis M, Piper K, Puett D, Rivenbark KM, Malchoff C (2014). Association of Hashimoto's thyroiditis with thyroid cancer. Endocr Relat Cancer.

[29] Ehlers M, Schott M (2014). Hashimoto's thyroiditis and papillary thyroid cancer: are they immunologically linked?. Trends Endocrinol Metab.

[30] Paparodis R, Imam S, Todorova-Koteva K, Staii A, Jaume JC (2014). Hashimoto's thyroiditis pathology and risk for thyroid cancer. Thyroid.

[31] Cunha LL, Marcello MA, Ward LS (2014). The role of the inflammatory microenvironment in thyroid carcinogenesis. Endocr Relat Cancer.

[32] Levy PS, Stolte K (2000). Statistical methods in public health and epidemiology: a look at the recent past and projections for the next decade. Stat Methods Med Res.

[33] Ding W, Zhou L, Bao Y, Zhou L, Yang Y, Lu B, Wu X, Hu R (2011). Autonomic nervous function and baroreflex sensitivity in hypertensive diabetic patients. Acta Cardiol.

[34] Chen Z, Liu J, Liang K, Liang W, Ma S, Zeng G, Xiao S, He J (2012). The diagnostic value of a multivariate logistic regression analysis model with transvaginal power Doppler ultrasonography for the prediction of ectopic pregnancy. J Int Med Res.

[35] Warner B, Misra M (1996). Understanding neural networks as statistical tools. Am Stat.

[36] Li H, Luo M, Zheng J, Luo J, Zeng R, Feng N, Du Q, Fang J (2017). An artificial neural network prediction model of congenital heart disease based on risk factors: a hospital-based case-control study. Medicine.

